# Photodynamic Therapy for Inflammatory and Cancerous Diseases of the Intestines: Molecular Mechanisms and Prospects for Application

**DOI:** 10.7150/ijbs.87492

**Published:** 2023-09-04

**Authors:** Beiying Deng, Kunpeng Wang, Lilong Zhang, Zhendong Qiu, Weiguo Dong, Weixing Wang

**Affiliations:** 1 Department of Gastroenterology, Renmin Hospital of Wuhan University, Wuhan, China.; 2 Department of General Surgery, Renmin Hospital of Wuhan University, Wuhan, China.

**Keywords:** photodynamic therapy, inflammatory bowel diseases, colorectal cancer, reactive oxygen species, immunology, nanomedicine

## Abstract

Photodynamic therapy (PDT) is a minimally invasive treatment that effectively targets cancer and inflammatory diseases. It has gained recognition for its efficacy, low toxicity, and potential for repeated use. Colorectal cancer (CRC) and inflammatory bowel diseases (IBD), including Crohn's disease (CD), and ulcerative colitis (UC), impose a significant burden on global intestinal health, with increasing incidence and prevalence rates. PDT shows promise as an emerging approach for gastrointestinal disease treatment, particularly IBD and CRC. Extensive preclinical research has demonstrated the safety and effectiveness of PDT for IBD and CRC, while clinical studies are currently underway. This review provides an overview of the underlying mechanisms responsible for the anti-inflammatory and anti-tumor effects of PDT, offering insights into the clinical application of PDT in IBD and CRC treatment. It is expected that this review will serve as a valuable reference for future research on PDT for CRC and IBD, contributing to advancements in the treatment of inflammatory and cancerous diseases of the intestines.

## 1. Introduction

Photodynamic therapy (PDT) is a highly effective and minimally invasive treatment for cancer and inflammatory diseases, known for its recognized efficacy, low toxicity, and suitability for repeated administration 1. The procedure involves the application of a photosensitizer (PS) to the targeted area, followed by exposure to specific light 2. The PS selectively targets rapidly proliferating cells, making it a versatile and effective treatment option for various medical conditions 3, 4.

PDT comprises three essential components: excitation light, PS, and oxygen. Table S1 summarized the types of PS and its application in various medical conditions. The choice of PS type and its application in different conditions depends on factors like disease type and stage, tumor location, specific PS properties, and treatment objectives. When a PS is exposed to light of a specific wavelength, it undergoes two types of photochemical reactions that lead to the generation of reactive oxygen species (ROS) 5. In Type I reactions, the PS transfers energy to biomolecules while in the T1 excited state, causing a transfer of hydrogen or electrons between the photosensitizer and substrate. Subsequently, interacting with oxygen molecules produces superoxide anion radicals, which generate ROS within the cells. Conversely, in Type II reactions, the PS is excited to the triplet state, and its energy directly transfers to ground-state oxygen molecules, resulting in the production of highly oxidizing singlet oxygen 1.

PDT can be categorized into High-dose (HDPDT) and low-dose (LDPDT) based on their efficiency in producing ROS, which is primarily influenced by the power and duration of light exposure 6, 7. HDPDT can damage cellular structure and function, leading to an anti-vascular effect, and is well-suited for tumor treatment 8-10. Importantly, elevated levels of ROS can lead to direct photo-damage of proteins, lipids, and other molecules within the PS area, resulting in cell necrosis and apoptosis. Additionally, ROS can cause damage to lysosomes or endoplasmic reticulum, potentially inducing autophagy or ferroptosis 11-13. Given the potential accumulation of the PS in the adjacent healthy tissues, HDPDT may inadvertently cause harm to normal cells in the intestine while targeting tumor cells 2, 14, 15. LDPDT shows notable efficacy in preventing mucosal damage 16, and demonstrates excellent immunomodulatory effects 17. Moreover, it regulates various intestinal bacterial strains and promotes neovascular closure. The successful application of LDPDT in treating inflammatory diseases highlights its favorable safety profile for therapeutic use. 7, 17-19.

Inflammatory bowel disease (IBD), encompassing ulcerative colitis (UC), Crohn's disease (CD), and colorectal cancer (CRC), represents a significant burden on global intestinal health, with increasing incidence and prevalence rates 20-22. Importantly, IBD is recognized as one of the contributing factors to the development of CRC 23. IBD-related colorectal cancer (CAC) represents approximately 2% of the total annual mortality from CRC. However, the annual mortality rate among individuals with IBD is significantly higher, ranging from 10% to 15%. Moreover, CAC patients tend to be diagnosed at a younger age compared to those with sporadic CRC, and their 5-year survival rate is 50% 24. PDT, being one of the established pillars of cancer treatment, is recommended for various types of tumors 25, and has proven to be an effective palliative treatment for advanced CRC 26, 27. Recent studies have provided evidence of PDT's ability to attenuate IBD through the downregulation of pro-inflammatory cytokines 7, regulation of intestinal microbiota 18, and modulation of miRNAs 16. The safety and effectiveness of PDT have been extensively demonstrated in preclinical research for IBD and CRC, and clinical studies are currently underway 28-30. The tubular structure of the intestine allows for precise treatment of intestinal lesions through endoscopic guidance. This significantly mitigates the limitations imposed by PDT's restricted penetration depth, leading to enhanced treatment efficacy 31, 32.

Hence, PDT shows immense promise as a novel approach in the treatment of gastrointestinal diseases, notably IBD and CRC. This review aims to comprehensively explore the mechanisms of PDT in managing inflammatory and neoplastic conditions of the intestines, providing valuable insights to promote its clinical implementation for IBD and CRC management.

## 2. Advantages of PDT in intestinal-related inflammatory and cancerous diseases

The management of IBD is multifaceted, typically involving prolonged administration of multiple medications and, in some cases, surgical intervention 33. IBD is a chronic condition without a definitive cure, placing patients at risk of various complications such as intestinal bleeding, perforation, and obstruction. In severe cases, IBD can progress to colorectal cancer 34. In advanced stages, immunosuppressive drugs may be necessary, although they can induce toxic side effects and impose a financial burden on patients. Close collaboration between healthcare providers is imperative to optimize treatment outcomes 35. Biologic therapies, including tumor necrosis factor-α (TNF-α) antibodies, have been employed in IBD treatment since the 21st century, demonstrating notable advancements. Nevertheless, the efficacy of these drugs varies among individuals with IBD, with approximately one-third of patients exhibiting unresponsiveness. Consequently, a combination of biologic therapy and immunomodulators is often necessary. However, it is worth noting that studies have indicated an increased risk of severe infections and T-cell lymphomas associated with this treatment. The burden of IBD has been consistently rising in both Western and Asian nations 22. Consequently, there is a pressing need to explore innovative therapeutic approaches for IBD. Ideally, these novel treatment options should exhibit the following attributes: 1) Fast-acting treatment; 2) Broad-spectrum effects; 3) lack of systemic immunosuppressive effects; 4) ability to induce alterations in bacterial antigens; 5) anti-angiogenic effects; 6) favorable safety profiles; and 7) suitability for patients with IBD at all disease stages.

PDT offers several advantages, including minimally invasive procedures, dual-targeted killing, minimal toxicity and side effects, and the potential for repeated treatments 1. It fulfills the criteria for the aforementioned new treatment options for IBD 19. Numerous studies have showcased the considerable advantages of PDT in treating inflammatory diseases, including IBD 4, 36, 37. PDT can modify cell signal transduction, regulate cytokine generation, and modulate cell surface receptor expression, thereby preserving cell vitality and exerting anti-inflammatory properties 7, 17, 38. The combination of biologic therapy and immunomodulators represents an essential and indispensable approach for managing IBD, particularly in patients with more severe disease manifestations. Table S2 provides a comparison of the mechanisms of action and therapeutic effects between PDT and this combination therapy. PDT offers a localized and targeted approach with minimal systemic effects, while combination therapy aims to modulate the immune response and control inflammation. The choice between these treatments depends on the specific characteristics and severity of the individual's IBD. Nevertheless, we firmly believe that PDT holds greater potential as a future treatment method for IBD. Moreover, in Table 1, we provide a summary of studies on the successful mitigation of IBD using PDT. Despite notable progress in the diagnosis and treatment of CRC, limited understanding exists regarding the mechanisms through which IBD contributes to CRC and colorectal liver metastasis (CRLM) 39. Patients with CRLM, particularly those with diffuse liver metastases originating from CRC, frequently encounter unfavorable clinical outcomes, underscoring the necessity for the advancement of novel therapeutic approaches 40. Promisingly, PDT exhibits substantial potential for application in CRLM 27, 39, 41.

**Figure 1:**
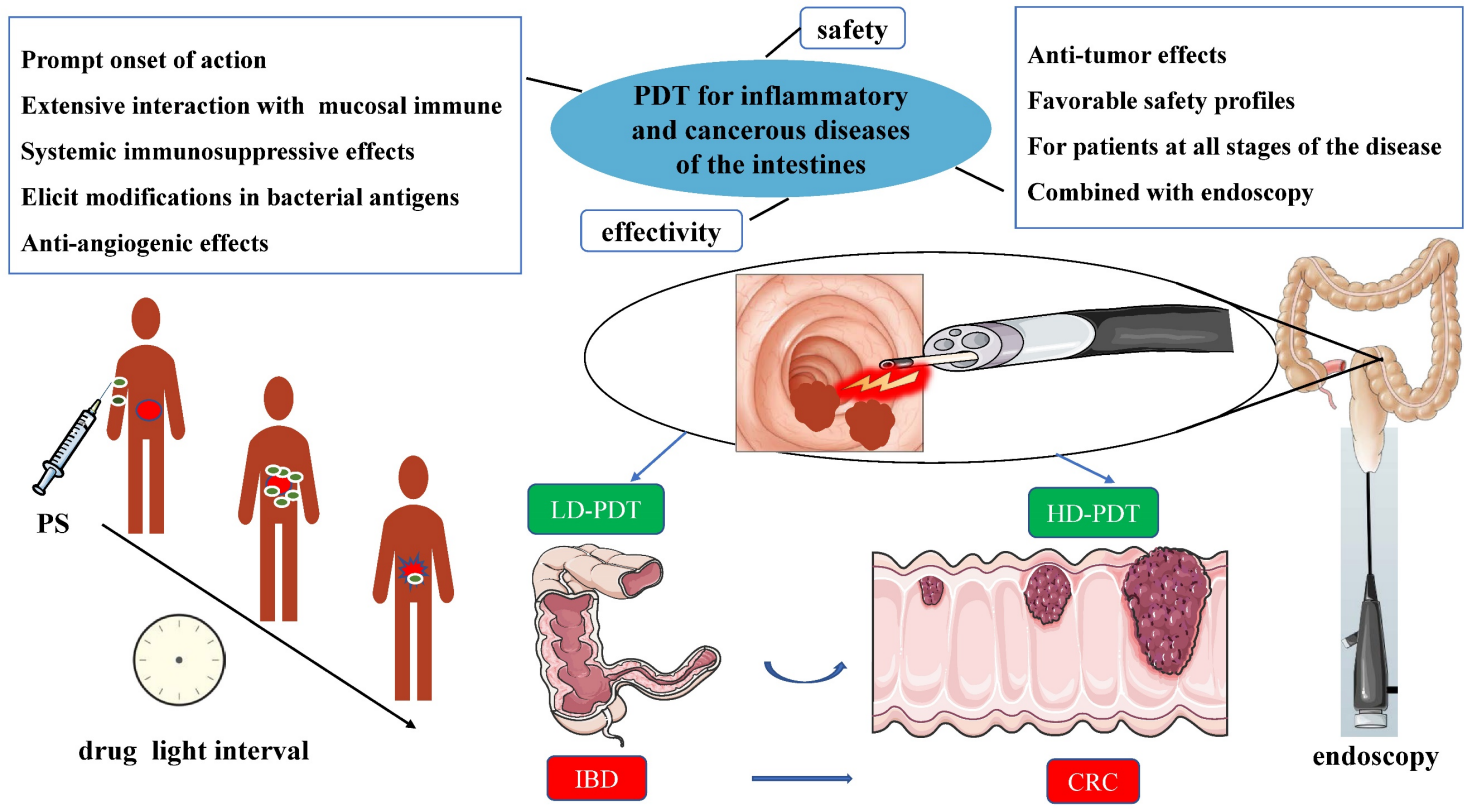
A schematic description of the application of PDT for inflammatory and cancerous diseases of the intestines.

The application of PDT in deep lesions is limited by the tissue penetration ability of the traditionally used near-infrared light source 42. However, the intestine's natural tubular structure and connection to the outside world through the anus provide a solution. By introducing a fiber optic cable through the anus, the intestinal mucosa can be effectively irradiated, addressing the issue of limited tissue penetration ability in PDT to a significant extent 16. PDT is particularly suitable for utilization in combination with endoscopes, such as colonoscopes, enabling accurate irradiation and treatment of intestinal lesions while providing direct endoscopic visualization. Moreover, Endoscopy serves as a primary diagnostic and evaluative tool for gastrointestinal diseases. 43. The direct visual observation and assessment of lesions, along with the ability to intervene, establish endoscopes as the gold standard 44. Hence, PDT offers significant advantages in the diagnosis and treatment of intestinal diseases, particularly in the context of IBD and CRC 31, 45. Figure 1 illustrates a schematic description of the application of PDT for inflammatory and cancerous diseases of the intestines.

## 3. The mechanisms of PDT in intestinal-related inflammatory and cancerous diseases

**Figure 2:**
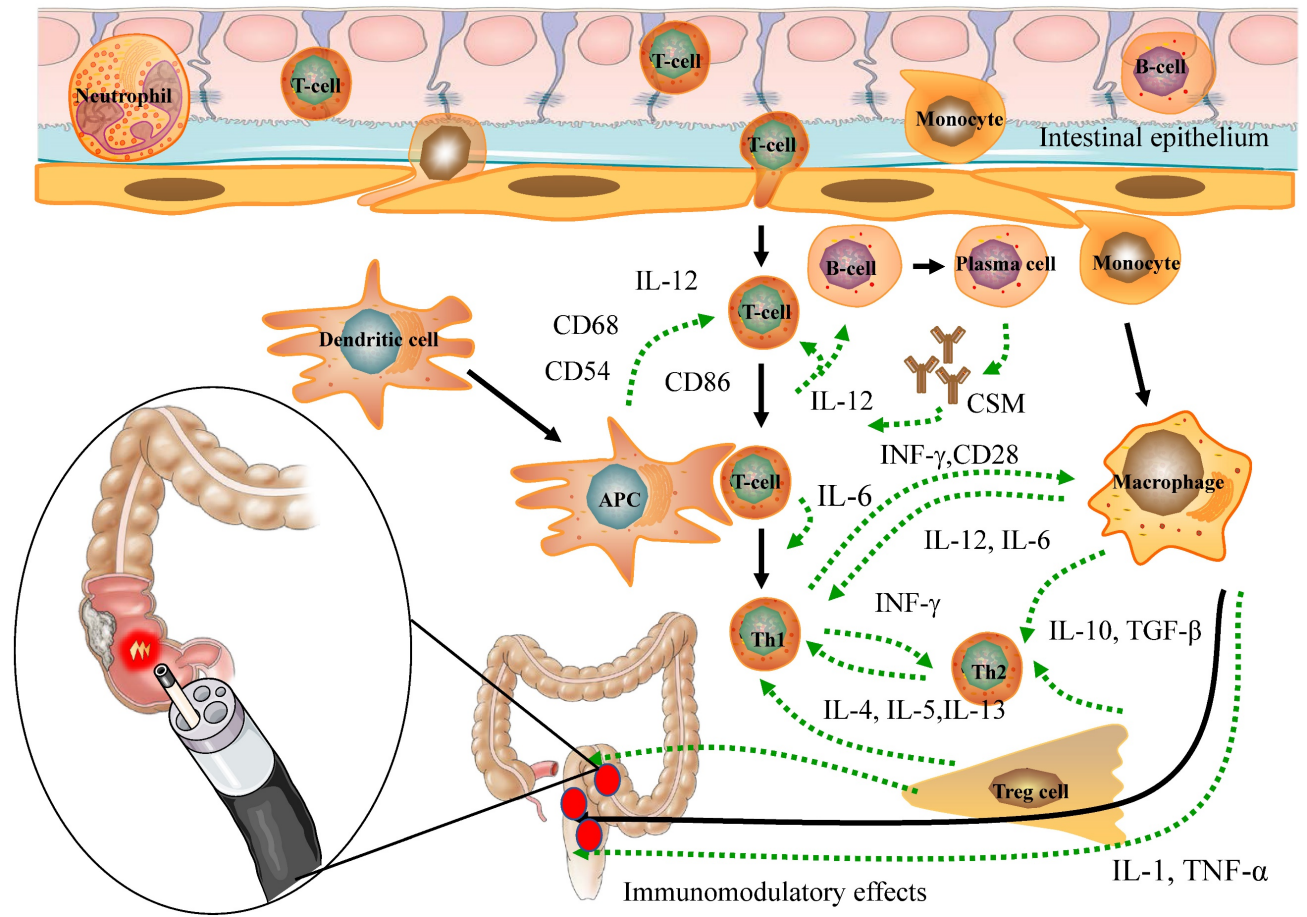
The immunomodulatory effects of PDT for inflammatory and cancerous diseases of the Intestines

**Figure 3:**
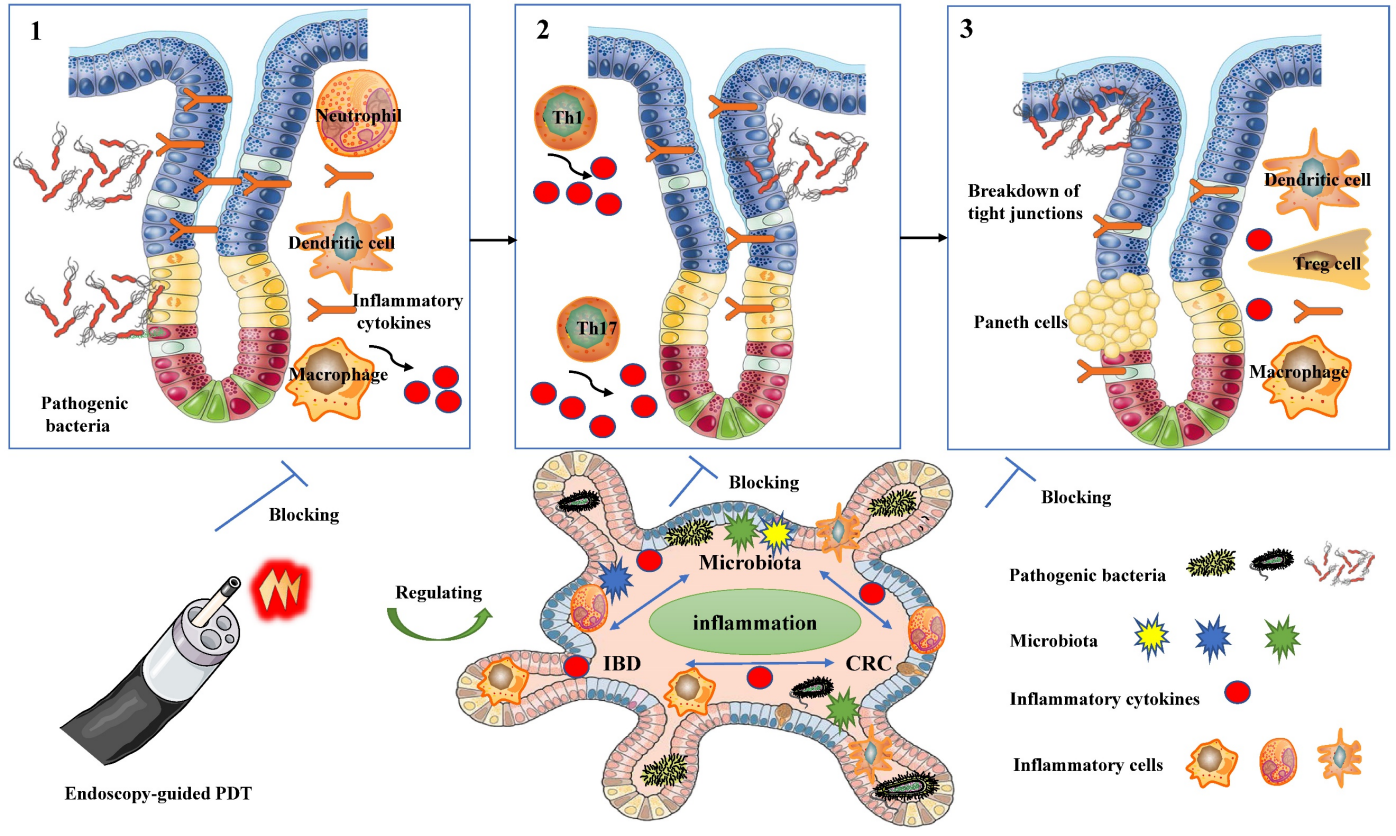
The modulation of microbiota homeostasis in PDT for inflammatory and cancerous diseases of the intestines.

**Figure 4:**
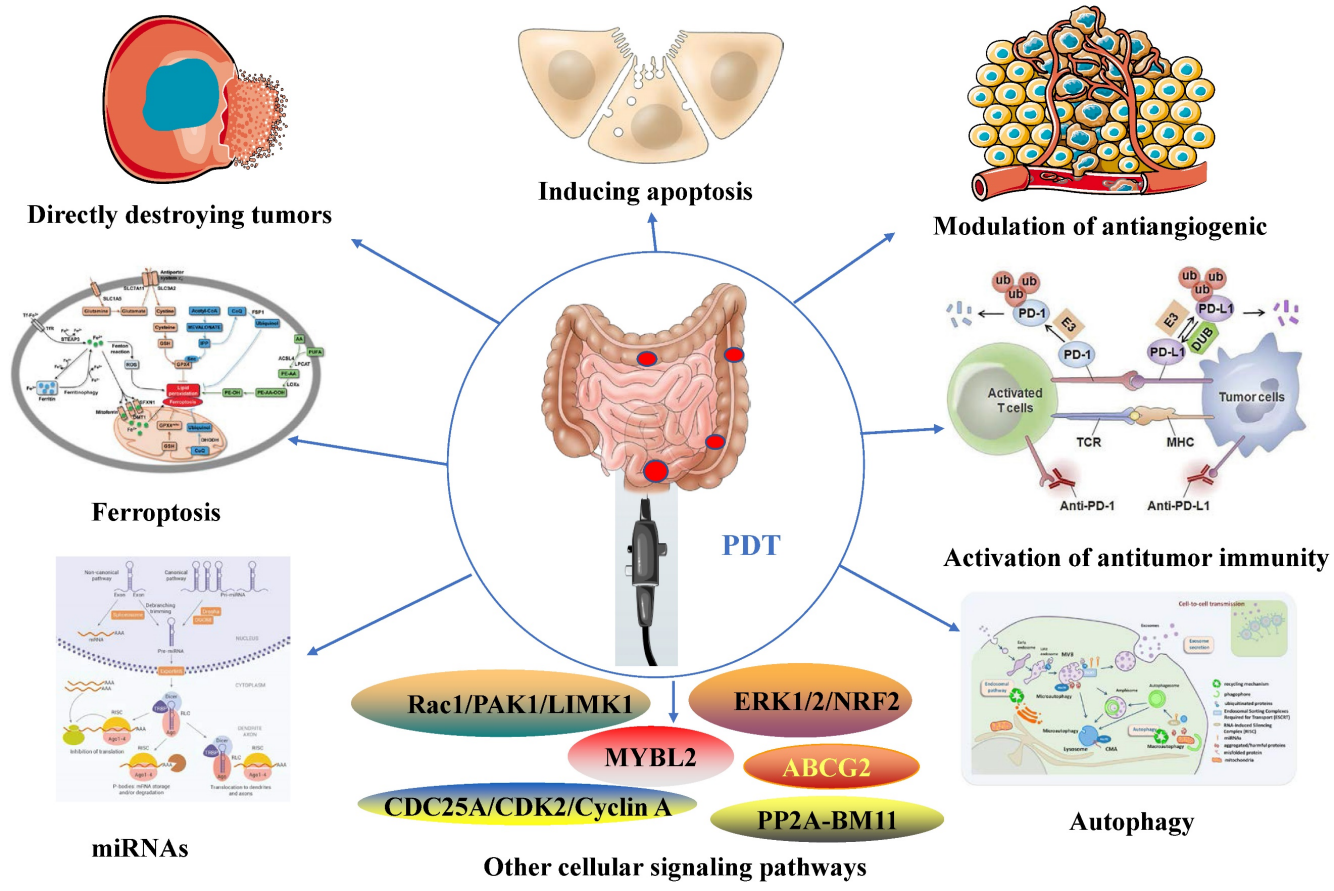
The anti-tumor effects of PDT for inflammatory and cancerous diseases of the intestines.

PDT possesses properties that enable the modulation of immune responses (Figure 2), regulation of microbiota homeostasis (Figure 3), and exhibition of anti-tumor effects (Figure 4).

### 3.1 Immunomodulatory effects of PDT

The influence of PDT on the immune response is intricate and contingent on diverse factors, encompassing the type, pharmacokinetics, and intracellular localization of PS, particularly within immune cells. Moreover, the effectiveness of ROS generation is influenced by factors like the wavelength, intensity, and duration of the activating light, which can impact PDT's regulation of the immune response 46. HDPDT is typically found to enhance the body's immune response. In particular, photo-induced cytotoxicity directly damages tumor cells, exposing tumor antigens, and thereby regulating tumor immune responses through inflammatory reactions for the treatment of colorectal cancer. CRC 47. On the other hand, LDPDT demonstrates immunomodulatory effects by altering immune cell functions, including the regulation of immune cell surface molecules and cytokine balance, thereby ameliorating IBD 7, 18 (Figure 2). In conclusion, both HDPDT and LDPDT exhibit remarkable immunomodulatory effects, laying a crucial foundation for the potential application of PDT in the management of inflammatory and neoplastic conditions of the intestines.

#### 3.1.1 Apoptosis of immune cells

During the application of HDPDT for CRC treatment, the PS accumulates in the mitochondria and, upon stimulation by specific light wavelengths, can induce apoptosis in host cells 48-50. Nonetheless, there is limited research on PDT-induced apoptosis of immune cells, with only a few studies demonstrating apoptosis in leukocytes, macrophages, and T cells following PDT treatment 51-53. The interaction between PS and immune cells is crucial in PDT-induced immune cell apoptosis. 4, 54, 55 Hydrophobic PS exhibits an affinity for binding with plasma proteins, especially low-density lipoprotein. Moreover, activated immune cells display higher PS absorption than resting immune cells 4, 54, possibly due to upregulated expression levels of lipoprotein receptors 53. As a result, even in LDPDT, a favorable immunomodulatory function is present. Consequently, the induction of immune cell apoptosis serves as one of the mechanisms by which PDT manifests its immunomodulatory effects. (Figure 2).

#### 3.1.2 Regulation of immune cell surface molecules

Numerous studies have demonstrated that PDT can modify the expression of diverse surface receptors on immune cells, consequently impacting the usual interaction between antigen-presenting cells (APCs) and T cells. This alteration hinders the effective activation of T cells by APCs following PDT treatment 55-57. Notably, PDT has been observed to downregulate the expression of specific adhesion molecules and major histocompatibility complex (MHC) molecules on immune cells 58. Furthermore, the expression levels of co-stimulatory molecules are crucial for T cell activation by PDT 59. Dendritic cells (DCs) are highly efficient APCs that activate immature T lymphocytes through abundant expression of MHC antigens, adhesion molecules, and co-stimulatory molecules. Moreover, they are essential producers of the pro-inflammatory cytokine IL-12, critically involved in regulating T-cell immune responses 57. PDT results in a substantial reduction of the expression levels of two types of MHC antigens and co-stimulatory molecules in mice with IBD 56. Furthermore, LDPDT has demonstrated the ability to suppress the production of pro-inflammatory cytokines while promoting the generation of anti-inflammatory cytokines. Additionally, has demonstrated immunomodulatory and anti-inflammatory effects in a murine arthritis model 60. Notably, both IBD and rheumatoid arthritis share immune-inflammatory mechanisms, and specific drugs are suitable for treating both disorders 61. The modulation of surface receptor expression by PDT holds significant implications for immune cell function and interactions. Through the alteration of these receptors, PDT can exert influence on immune cell signaling, antigen presentation, and immune responses. Taken together, PDT significantly modifies various surface receptors on immune cells, which in turn influences the interaction between APCs and T cells, ultimately impacting immune cell function and responses. This presents a promising therapeutic approach for conditions characterized by immune dysregulation, including IBD and CRC. (Figure 2).

#### 3.1.3 Modulation of cytokine balance

PDT modulates cytokine balance, thereby influencing immune cell function and interactions, and holds promise as a therapeutic approach for immune-related conditions 25. Studies have demonstrated that the administration of low-dose PS 5-ALA (15mg/kg) and exposure to light at a wavelength of 635nm, fluence of 10J/cm^2^, and power density of 100 mW/cm^2^ significantly reduced the secretion of pro-inflammatory cytokines IL-6, IL-17, and IFN-γ, as well as the number of CD4^+^ T cells in a murine model of T cell-mediated colitis 7. Moreover, the administration of low-dose PS Foslip (0.01mg/kg) and exposure to light at a wavelength of 652nm, fluence of 20 J/cm^2^, and power density of 100 mW/cm^2^ significantly decreased the expression of pro-inflammatory cytokines TNF-α, IL-1β, IL-12, and IFN-γ, while increasing the expression of the anti-inflammatory cytokine IL-10 in a mouse model of DSS-induced ulcerative colitis 18. Furthermore, the administration of low-dose PS 5-ALA (15mg/kg) and exposure to light at a wavelength of 630nm, fluence of 10J/cm^2,^ and power density of 300 mW/cm^2^ significantly downregulated the expression levels of pro-inflammatory cytokines IL-17a, TNF-α, and IL-12a 16.

In addition to effectively destroying malignant colorectal cancer tissue, PDT also exhibits anti-cancer activity by reducing the secretion of IL-6 and IL-10 in CRC cell lines 62. Furthermore, ALA-LDPDT has the potential to influence the progression and invasion of colorectal cancer cells. It decreases the secretion of IL-6 and IL-10 while increasing the concentration of IL-8. Notably, cell lines with a higher degree of malignancy exhibit higher concentrations of IL-8 63. Moreover, PDT regulates nitric oxide, IL-6, and TNF-α to enhance the cytotoxicity of tumor-associated non-resident macrophages in CRC 64.

Notably, the imbalance of cytokines can contribute to the development of cancer, including CRC, in chronic inflammatory diseases like IBD. Growing evidence suggests the involvement of various cytokines released by epithelial and immune cells in the development of CAC. Crucially, cyclooxygenase-2 (COX-2) and nuclear factor kappa B (NF-kB), are crucial genes responsible for regulating cytokine balance and mediating the intricate interplay between inflammation and cancer 65. Moreover, recent studies have shown that additional factors, such as the IL6/STAT3, IL22/STAT3, and IL23/STAT3/Th17 signaling pathways, contribute to the onset of CAC 66. Importantly, during the recovery period after PDT, tissue cells maintain the expression of IL-6 or Hyper-IL-6, leading to increased inhibition of proliferation and offering a more effective strategy for tumor control 67. These studies collectively demonstrate the significant role of PDT in modulating cytokine balance and its potential value in preventing and treating IBD, CAC, and CRC (Figure 2).

### 3.2 Modulation of microbiota homeostasis

The antibacterial efficacy and the potential of PDT for local infection treatment have been extensively validated over the past few decades 4. Recently, there has been a growing interest in the role of PDT in modulating microbiota homeostasis 18, 19. The delicate balance of the gut microbiome is of utmost importance in preserving intestinal health and influencing the progression of both IBD and CRC 68. Reinhard et al. were the first to investigate the use of LDPDT therapy in preventing the occurrence of dysbiotic microbiota in a CAC model 18. Research has suggested that Foslip-mediated PDT demonstrates effectiveness, safety, antimicrobial, anti-inflammatory, and anti-carcinogenic properties in IBD 18. The mechanism of PDT involves enhancing the resilience of the intestinal barrier through the repair of E-cadherin tight junctions and mucin-2 secretion, as well as reshaping the composition of the gut microbiota in a colitis-associated carcinogenesis model 18. The study showed that LDPDT did not directly affect bacterial symbiosis in mice, but there were significant differences in bacterial composition between the LDPDT treatment group and the disease control group.AOM/DSS mice exhibited lower phylodiversity and higher bacteroidaceae abundance in tumor-bearing animals. Conversely, LDPDT-AOM/DSS mice did not significantly differ from healthy controls, suggesting that LDPDT might modulate the microbiota homeostasis in IBD 18.

Moreover, a recent study reported that LD4-PDT facilitated the healing of colonic mucosa, regulated gut microbiota, and ameliorated clinical symptoms of UC 19. This study presents compelling evidence supporting the multifaceted effects of LD4-PDT, which include the regulation of inflammatory cytokine balance and modulation of gut microbiota composition. These combined actions result in the restoration of the colonic proteome in TNBS-induced colitis rats, leading to the effective alleviation of acute colitis 19. Mechanistically, the beneficial effects of LD4-PDT on colitis in rats may be attributed to its regulation of the AOC1 target 19. Interestingly, LD4-PDT restored the gut microbiota in UC model rats to a state similar to that of the control group, despite significant differences in the TNBS model group. The proportion of Bacteroidetes was higher but less abundant in TNBS model rats, resulting in higher Firmicutes/Bacteroidetes (F/B) values. Following LD4-PDT treatment, Bacteroidetes decreased, highly viscous microorganisms increased, and F/B values decreased, similar to the control group 19. These findings highlight the significant potential of PDT in modulating microbiota homeostasis.

Research conducted thus far has underscored the significant role of microbiota in shaping the immune system and maintaining the overall health of the host 68-70. On one hand, an imbalance in Helicobacter or bacteria that produce genotoxins can trigger inflammation and promote tumor growth 71. On the other hand, patients with IBD or CRC demonstrate distinct microbial compositions compared to healthy individuals. These findings align with observations made in mouse models of IBD and CRC 72, indicating that dysbiosis of the microbiota is crucial in the development of these diseases 69 However, the relationship between the microbiota, inflammation, IBD, and CRC remains complex and not yet fully understood. Inflammation serves as a critical link in this relationship and is essential for dysbiosis and the development of CRC. Both dysbiosis of the microbiota and the development of CRC can trigger sustained inflammation in the intestine. Consequently, it remains uncertain whether dysbiosis of the microbiota in IBD or CRC is the cause, consequence, or a result of causal interaction 73. Nevertheless, three key factors cannot be disregarded in the intricate network of chronic inflammation in the gut microbiota and IBD and CRC: (1) colonization and invasion by pathogenic bacteria, which elicit inflammation by evading host defenses; (2) dysbiosis resulting from various factors, leading to chronic inflammation involving Th1 and Th17 cells; and (3) impaired host immune responses and immune regulation, resulting in inflammation due to increased antigen exposure and dysfunction of regulatory T cells or anti-inflammatory cytokines 69, 73. Additionally, accumulating evidence suggests that the gut microbiota influences immunogenic cell death (ICD) and the anti-tumor effects of PDT 68, 69. Taken together, PDT is crucial in modulating microbiota homeostasis in inflammatory and cancerous diseases of the intestines, demonstrating promising potential as a therapeutic approach for managing these conditions. (Figure 3).

### 3.3 Anti-tumor effects of PDT

PDT has been widely verified for its potent anti-tumor properties 74. The localized nature of PDT allows for precise targeting of tumor cells, minimizing harm to healthy tissues. Furthermore, it has the potential to enhance the effectiveness of other treatment modalities 75. PDT shows promise in the treatment of various tumor types, including CRC, and continues to be an active area of research and clinical development 47. In this section, we provide a comprehensive overview of the molecular mechanisms underlying the anti- CRC effects of PDT. (Figure 4).

#### 3.3.1 Directly destroying tumors and inducing apoptosis

PDT generates ROS that directly damages tumor cells, resulting in cellular apoptosis and necrosis 25. Apoptosis is a physiological process that eliminates damaged or unwanted cells in the body. Disruption of this process can lead to uncontrolled cell growth and the formation of cancer. Inducing apoptosis in cancer cells is a commonly employed strategy for cancer treatment 76. Extensive research has focused on inducing apoptosis in CRC cells through PDT 77. PDT markedly increases the extent of apoptosis in CRC cells 48. Moreover, PDT activates apoptosis pathways mediated by mitochondria, endoplasmic reticulum (ER), and lysosomes 49, 78. Additionally, the involvement of apoptotic pathways such as p38 MAPK/caspase9 50, Bax 79, and p53 80 is also crucial in this process. In conclusion, PDT triggers apoptosis in CRC cells through the activation of multiple apoptotic pathways and significant changes in gene expression, as illustrated in Table 2.

#### 3.3.2 Modulation of antiangiogenic

PDT induces the destruction of tumor blood vessels through the production of ROS from the PS. This leads to vasoconstriction, rupture, and thrombosis, ultimately causing tumor ischemia and necrosis 81, 82. The extent of vascular damage following PDT is influenced by various factors, including the pharmacokinetics of the PS, the intensity and fluence of the excitation light, and the time interval between PS injection and light excitation (drug-light interval). A short drug-light interval primarily results in acute vascular damage, while a long drug-light interval induces greater oxidative damage to the tumor tissue 83. Moreover, the oxidative stress response following PDT is influenced by the TME, which intricately regulates multiple molecular signaling pathways, leading to the promotion of vascular endothelial growth factor (VEGF) expression 84, This, in turn, impacts the antiangiogenic effect of PDT. VEGF is a critical regulatory factor in the process of angiogenesis during tumor progression. As a tumor grows, it secretes growth factors like VEGF, which stimulate endothelial cells and facilitate extracellular matrix restructuring, leading to the formation of new blood vessels. This process is necessary to maintain an adequate oxygen and nutrient supply for further tumor growth 85.

Contradictory findings exist regarding the effect of PDT on VEGF secretion by other tumor cells. However, studies investigating PDT treatment of CRC consistently report positive outcomes 86, 87. Kawczyk et al. conducted an *in vitro* study to examine the impact of PDT on VEGF secretion by CRC cells under aerobic conditions 86. Their findings revealed that 5-ALA-PDT significantly reduced VEGF secretion in the metastatic CRC SW620 cell line, while the non-metastatic CRC SW480 cell line maintained its VEGF secretion ability unchanged. Moreover, regardless of PDT intervention, the SW620 cell line exhibited significantly higher VEGF secretion capacity than SW480, aligning with recent studies 87. Therefore, PDT employs a multifaceted mechanism to modulate CRC angiogenesis, involving both direct damage to tumor vasculature and the regulation of oxidative stress response and VEGF expression within the tumor microenvironment. (Table 2).

#### 3.3.3 Activation of antitumor immunity

PDT has been demonstrated to activate the immune system and induce ICD in tumor cells through the production of ROS from the PS. This activation promotes antigen presentation and T cell-mediated immune responses, thereby enhancing antitumor immune responses 88. Additionally, PDT can increase the expression of tumor antigens, activate immune cells, and deplete T regulatory cells (Tregs), leading to the induction of long-term survival and memory immune responses 89-91. In a study by Mroz et al., it was observed that PDT induces systemic, antigen-specific anti-tumor immunity in CRC BALB/c mouse models expressing a tumor-specific antigen 92. Furthermore, the immune response mediated by PDT is associated with Treg depletion, which enhances the immune response and triggers long-term survival and memory immune responses 93. These findings contribute to the understanding of the complex regulatory mechanisms involving PDT, Tregs, the tumor microenvironment, and different types of tumor antigens. Additional evidence has confirmed the synergistic therapeutic effects of combining NCP@pyrolipid-PDT with anti-PD-L1 therapy, resulting in the inhibition of primary CRC growth and suppression of distant metastasis 94. Similarly, the combination of Fe-TBP-PDT with anti-PD-L1 treatment can induce synergistic therapy involving CD4^+^ and CD8^+^ cytotoxic T cells for CRC treatment 95. Likewise, UCNP-Ce6-R837-PDT triggers dendritic cell maturation and cytokine secretion to stimulate immune responses. When combined with cytotoxic T-lymphocyte-associated protein 4 (CTLA-4) checkpoint inhibitors, it inhibits Treg cell activity and exhibits a powerful synergistic therapeutic effect. This combined treatment not only eliminates primary CRC tumors and inhibits distant metastasis but also prevents recurrence 96. Notably, Li et al. reported an innovative approach that combines PDT with immunotherapy using a PS specifically targeting EGFR. This approach demonstrates precise targeting for CRC with remarkable inhibitory effects and no recurrence after treatment 97. Moreover, the PS SPDMCN is specifically activated in the CRC tumor microenvironment, releasing an immune modulator (DMC) to enhance the effect of PDT 98. Furthermore, PDT has been shown to inhibit the proliferation of multiple CRC cell lines by inducing ICD 88. Collectively, these studies provide a foundation for the application of PDT combined with immunotherapy in cancer treatment (Table 2) 95, 99-101.

#### 3.3.4 Modulation of cellular signaling pathways

PDT can disrupt multiple cellular signaling pathways, including autophagy 102, ferroptosis 103, miRNA 104, and inflammatory signaling pathways 105. These regulatory effects have a direct impact on the viability and proliferation of cancer cells, ultimately contributing to tumor regression.

##### 3.3.4.1 Autophagy

Autophagy is a metabolic process that involves the degradation and recycling of unnecessary or harmful components within cells, providing the energy and nutrients necessary for cell survival. Under normal conditions, autophagy contributes to maintaining cellular homeostasis, preventing DNA damage, and inhibiting abnormal cell proliferation 106-108. The role of autophagy in cancer is complex and has been shown to exhibit both pro-tumorigenic and anti-tumorigenic effects, depending on various factors including the type of cancer, tumor microenvironment, metabolic status of tumor cells, and regulatory mechanisms of autophagy pathways. Furthermore, the pro-tumorigenic and anti-tumorigenic effects of autophagy can interchange 109-111. Nonetheless, the importance of autophagy in suppressing CRC through PDT is widely recognized. PDT can induce autophagy in CRC cells, leading to their death. Additionally, PDT can enhance its cytotoxic effects on CRC cells by inhibiting autophagy 112. Therefore, combining PDT with autophagy inhibitors holds promise as a potential approach for treating CRC 113.

It has been reported that blocking autophagy can enhance the susceptibility of PROM1/CD133+ cells to apoptosis induced by PDT, while also reducing the tumorigenic potential of colorectal cancer stem cells (CSCs) 113. A combination therapy targeting p38MAPK and autophagy, in conjunction with PDT, may offer an effective treatment for CRC. This is because inhibiting p38MAPK enhances the efficacy of PDT, while autophagy provides protection against photokilling 114. Ziółkowska et al. conducted an initial analysis of the impact of PDT on the expression of autophagy-related proteins Beclin-1, Atg7, and LC3, which has deepened our understanding of the relationship between autophagy and PDT 115. Similarly, recent studies have confirmed that the combination of PDT with autophagy enhances the inhibitory effect on CRC 13, 112, 116-119. Furthermore, PDT triggers autophagy as a survival mechanism, and the activation of the novel HIF-1α/VMP1/autophagic pathway may shed light on the resistance of CRC cells to PDT 120. Although it is generally believed that inhibiting the expression of the key autophagy substrate p62 can enhance the anti-tumor effect, Kim et al. have shown that overexpressing p62 can increase the effectiveness of PDT. Additionally, they found that CRC cell lines with p62 knocked out exhibit decreased sensitivity to PDT 121. Inhibiting autophagy can significantly reduce the efficacy of PDT-induced CRC cells by deactivating the ROS/JNK signaling pathway 122. Corporately, these studies provide a compelling rationale for combining PDT with autophagy inhibitors for the treatment of CRC (Table 2).

##### 3.3.4.2 Ferroptosis

Ferroptosis is a regulated cell death mechanism that triggers the overproduction of lipid-based ROS by inhibiting the cystine/glutamate antiporter system and the synthesis of glutathione (GSH) 123. Uniquely, ferroptosis increases the cellular labile iron pool (LIP), leading to the continual generation of O_2_ through the Fenton reaction of H_2_O_2_ and Fe (III) 124.

Emerging cancer therapy strategies based on ferroptosis have garnered interest 125, 126. Increasing the concentration of ROS and O_2_ in the tumor microenvironment (TME) can enhance the effectiveness of PDT. Fortunately, the characteristics of ferroptosis cells mentioned earlier precisely match what is needed to enhance PDT 127. There is evidence that PDT can function as a ROS generator, thereby triggering the Fenton reaction. This may intensify the initiation of ferroptosis and increase the effectiveness of PDT in anticancer treatment 127-129. Inducing ferritin production through PDT could serve as a promising alternative strategy to enhance the efficacy of CRC treatment, especially considering the immunological properties of ferritin 130. A better understanding of the synergistic interactions between PDT and ferritin may lead to more effective anti-CRC therapy by overcoming resistance to other cell death mechanisms.

Zhou et al. presented a promising treatment for CRC involving copper-cysteamine nanoparticles (Cu-Cy)-mediated PDT to induce ferroptosis in CRC cells. Crucially, *in vivo*, experiments using an HCT15 tumor-bearing mouse model provided additional confirmation of the superior antitumor efficacy of Cu-Cy-PDT 12. Additionally, iron administration was found to enhance the anticancer effect of Photolon-based PDT in a CT26 CRC mouse model 131. Iron-induced oxidative cellular damage might have contributed to the observed increase in the anticancer effect of PDT (Table 2) 131. These studies suggest that PDT-induced ferroptosis holds promising potential as an avenue to enhance the efficacy of CRC treatment.

##### 3.3.4.3 MiRNAs

MiRNAs exert substantial influence over gene expression and govern diverse cellular processes, encompassing proliferation, apoptosis, and differentiation 132. Recent studies have demonstrated their involvement in the mechanism of action of PDT 133. Specifically, miR-21 upregulation is triggered by PDT in cancer cells, and its inhibition enhances PDT-induced cell death 134. Additionally, miRNAs regulate TME, which is critical for PDT efficacy. For example, miR-155 modulates the TME by regulating the immune response to PDT 135. These findings suggest that targeting miRNAs can enhance the efficacy of PDT and overcome resistance to therapy.

A study conducted by Liu et al. revealed the role of miR-124 and iASPP in mediating resistance to PDT in CRC cells with p53 mutations or deletions. This finding holds significant importance in enhancing the effectiveness of PDT and optimizing treatment strategies 104. Furthermore, the long non-coding RNA LIFR-AS1 modulates the resistance of CRC cells to PDT by regulating miR-29a, which in turn regulates TNFAIP3 as a downstream target of miR-19a. This discovery highlights the potential of targeting LIFR-AS1 as a strategy to overcome PDT resistance in CRC 136. Additionally, PDT reduces the expression of miR7112-3p in CRC cell lines, thereby mitigating the inhibition of PERK. This results in increased PERK expression and induction of apoptosis in CRC cells through endoplasmic reticulum stress 78. Moreover, recent studies have demonstrated the critical involvement of the c-Myc/NEAT1 axis in mediating the therapeutic efficacy of PDT in CRC through the miR-124/iASPP/p53 pathway.^137^ (Table 2). To conclude, the therapeutic mechanism of PDT relies on the involvement of miRNAs. By targeting miRNAs, it is possible to enhance the effectiveness of PDT and surmount treatment resistance.

#### 3.3.4.4 Other signaling pathways

The mechanisms that trigger signaling pathways via PDT may depend on various factors, including cell type, metabolic characteristics 138, subcellular localization of the PS 139, and PDT treatment intensity 140. PDT for CRC is governed by the intricate involvement of multiple signaling pathways in its regulatory processes. Modulating NRF2 has been shown to enhance PDT sensitivity in various cancer cells, including CRC 105. Moreover, the activation of the ERK1/2 pathway, mediated by oxidative stress, critically contributes to the positive regulation of HIF-1 transcriptional activity after PDT. Effective strategies to mitigate PDT-induced upregulation of HIF-1 include ROS clearance and inhibition of the MEK/ERK pathway 141. The Rac1/PAK1/LIMK1/cofilin signaling pathway also exerts a notable impact on inhibiting CRC cells during PDT 142. Additionally, PDT triggers a toxic response in CRC cells by promoting the PP2A-mediated ubiquitination and degradation of BMI1^143^. Furthermore, the CDC25A/CDK2/Cyclin A pathway and the mitochondrial apoptosis pathway participate in the induction of cell cycle arrest and apoptosis in CRC cells following PDT 144. MYBL2 loss in CRC cells activates NF-κB, resulting in the upregulation of ABCG2 and subsequent inhibition of the CRC response to PDT 145. In conclusion, the molecular mechanisms and signaling pathways involved in the PDT treatment of CRC are complex and necessitate further comprehensive investigation (Figure 4).

## 4. Clinical Application of PDT in intestinal-related inflammatory and cancer diseases

Table 3 comprehensively reviews clinical trials and case reports investigating the use of PDT in intestinal-related inflammatory and cancerous diseases. In a clinical trial of six patients with advanced rectal cancer, Photofrin II-mediated PDT demonstrated remarkable tumor destruction in two patients. Moreover, one patient experienced relief from pain and obstruction symptoms. Importantly, the study reported no significant adverse effects, such as severe bleeding, sepsis, or perforation.^146^. Another study demonstrated the effectiveness of adjuvant intraoperative photodynamic therapy (AIOPDT) in reducing postoperative recurrence rates among patients with CRC 147. Furthermore, PDT holds promise as a potential technique for treating small gastrointestinal tumors or tumors in patients unsuitable for surgery 148-151. Importantly, PDT has been demonstrated to be a safe and effective treatment option for unresectable CRLM 152. Furthermore, Zhang et al. reported a successful case of PDT treatment for R1 rectal cancer 27. Regrettably, there is currently a lack of available clinical research reports investigating the utilization of PDT for the treatment of IBD (Table 3). Hence, the clinical application of PDT in intestinal-related inflammatory and cancerous diseases still faces significant challenges and requires further advancements before widespread adoption can be achieved.

## 5. Challenges and Prospects

### 5.1 Challenges

PDT demonstrates the ability to selectively target and affect diseased cells, including cancerous cells and inflamed tissues while sparing healthy cells. This selectivity significantly reduces damage to healthy tissues and minimizes side effects 153. Moreover, PDT qualifies as a minimally invasive treatment option that can be administered endoscopically. This characteristic enables direct application to the affected area, resulting in localized treatment and faster recovery times 43. Therefore, PDT shows promise as an effective approach for treating both inflammatory and cancerous diseases affecting the intestines 4, 47. However, this therapeutic method presents several challenges 154. The effectiveness of PDT heavily depends on the selective uptake of the photosensitizing agent by target cells 25. Achieving specific targeting can be challenging, particularly in cases involving diffuse or widespread lesions 155. Moreover, PDT protocols and parameters necessitate standardization and optimization for various diseases 156, 157, including inflammatory and cancerous conditions of the intestines 16. Further research is necessary to determine the optimal photosensitizers, light dosimetry, and treatment regimens for maximum efficacy 158. Furthermore, PDT is a complex and specialized therapy that requires specific equipment and expertise. The cost and availability of PDT may hinder its widespread adoption, especially in resource-limited settings 159. Although PDT is generally well-tolerated, it can still result in side effects and complications, such as skin photosensitivity, pain during light activation, and potential damage to adjacent healthy tissues if not precisely targeted 11.

### 5.2 Prospects

The advancements in science and technology, coupled with a growing understanding of PDT among researchers and practitioners, have significantly contributed to overcoming challenges 160. The potential of utilizing PDT in the treatment of IBD and CRC continues to exhibit promising prospects. In this article, we discuss several potential directions for future breakthroughs.

#### 5.2.1 Combination therapy

PDT can be combined with other treatment modalities, such as chemotherapy (e.g., 5-FU 161, doxorubicin 74, oxaliplatin 100), immunotherapy (e.g., anti-PD-L1 antibody 162-164, CTLA-4 inhibitors 101), targeted therapy (e.g., bevacizumab 165, Cetuximab 166), or small molecule inhibitors (e.g., inhibitors of COX-1 and COX-2^167^, proteasome inhibitors^168^, AKT inhibitors^169^, autophagy inhibitors^113^, ^170^), to enhance the overall therapeutic effect. This multimodal approach holds the potential to improve treatment outcomes, especially in cases where diseases have advanced or exhibit resistance to conventional therapies.

#### 5.2.2 Nanomedicine and Carriers in PDT

The application of nanomedicine is instrumental in augmenting the efficacy of PDT by improving the delivery, targeting, imaging, and enabling combination therapies 171. Nanoparticles effectively encapsulate PS, providing protection and ensuring efficient and sustained delivery to the intended site 172. Surface-functionalized nanoparticles can bind to cancer cell receptors, enhancing selectivity and reducing side effects 173. Moreover, nanoparticles incorporating imaging agents enable real-time visualization and precise treatment planning 45. Additionally, they can carry multiple components, such as chemotherapeutic drugs and immune checkpoint inhibitors, leading to enhanced therapeutic effects 162, 174. The integration of therapy and diagnostics in theranostic platforms further optimizes the outcomes of PDT 175, 176. Ultimately, nanomedicine leverages the unique properties of nanoparticles to improve the efficacy and precision of PDT. Importantly, the hypoxic characteristics of the TME can adversely impact the therapeutic efficacy of PDT. However, MnO_2_ nanoparticles can react with endogenous H_2_O_2_ and generate oxygen. Therefore, by encapsulating the PS within the lipid bilayer of liposomes and subsequently coating them with MnO_2_ nanoparticles, PDT can be enhanced in hypoxic tumor regions 177. Furthermore, Cu-Cy nanoparticles containing Cu_1_^+^ ions can participate in heterogenous Fenton-like activity under acidic conditions, leading to a significant increase in the levels of hydroxyl radicals within cancer cells. This mechanism enables the effective mitigation of low oxygen conditions within the tumor microenvironment, further enhancing the therapeutic outcomes of PDT 178.

The successful implementation of PDT heavily relies on efficiently delivering PS to target cells in complex biological environments. Therefore, developing innovative strategies to enhance PS delivery is of paramount importance 179. To overcome the challenges associated with direct PS administration, researchers have explored carrier-mediated delivery systems such as liposomes 180, aggregation-induced emission (AIE) luminogen hybrid nanovesicles 181, and extracellular vehicles (EVs) 182 to facilitate PS transport to target cells. Liposomes, which are spherical vesicles composed of lipid bilayers closely resembling biological membranes, serve as versatile carriers for PS 183. They can encapsulate PS within their hydrophobic core or integrate it into their lipid bilayers. The high surface area-to-volume ratio of liposomes enables efficient drug loading and controlled release. Additionally, surface modifications enhance the targeting specificity and cellular uptake of PS 184. AIE luminogen hybrid nanovesicles combine the photostability and fluorescence of AIE luminogens with the drug delivery capabilities of nanovesicles 185. These carriers exhibit excellent stability, high permeability, and tunable surface properties, making them promising vehicles for efficient PS delivery 186. EVs, naturally released vesicles involved in intercellular communication, have garnered interest as carriers for PS delivery 187. These vesicles can be loaded with PS and functionalized to achieve targeted delivery. Due to their inherent biocompatibility, low immunogenicity, and ability to traverse biological barriers, EVs are attractive candidates, especially for PS delivery in complex intestinal conditions 188.

#### 5.2.3 Novel Excitation Conditions for PDT

PDT exhibits promise in cancer treatment. Nonetheless, to augment its efficacy, researchers have investigated the integration of PDT with other modalities, such as X-ray, microwave, and sonodynamic therapy.

X-ray radiation possesses the capability to penetrate deep into tissues, rendering it a potential synergistic partner for PDT 189, 190. Multiple studies have explored the amalgamation of X-ray radiation with PDT, aiming to enhance tumor targeting and treatment outcomes 191,192,193. It shows potential in treating IBD and CRC. In IBD, X-inducers selectively accumulate in inflamed tissues and, upon X-ray activation, produce ROS, which reduces inflammation 190. In CRC, X-inducers can be targeted to cancer cells, generating ROS and inducing tumor cell death 193. X-PDT offers targeted therapy with reduced side effects and can be combined with other treatments 191. Importantly, PDT allows for multiple repetitions, if necessary, without the risk of cumulative toxicity. This feature proves particularly beneficial in managing chronic conditions, such as IBD, that require long-term treatment 7.

Microwave hyperthermia has been investigated in conjunction with PDT to enhance its therapeutic effects 194. Microwave-induced hyperthermia can augment blood flow, oxygen supply, and the permeability of tumor blood vessels, which can facilitate the delivery of PS to tumor cells and intensify the generation of reactive oxygen species during PDT 194. The combination of microwave hyperthermia and PDT has demonstrated improved tumor control and heightened treatment response in specific studies 12.

Sonodynamic therapy involves the use of ultrasound in combination with a sonosensitizer to induce the production of reactive oxygen species and achieve tumor destruction 182. When combined with PDT, sonodynamic therapy can further enhance the cytotoxic effects on tumor cells 195. Ultrasound waves can enhance the cellular uptake of PS and improve its localization within the tumor 196. Moreover, ultrasound-induced cavitation can promote the release of PS from carriers and enhance its intracellular delivery 197. The combination of sonodynamic therapy and PDT has shown the potential in overcoming the limitations of conventional PDT and improving its therapeutic outcomes 198.

## 6. Conclusions

PDT holds promise as a minimally invasive and effective treatment for CRC and IBD. Extensive preclinical research provides strong support for its safety and efficacy, while ongoing clinical studies further validate its potential. This review offers valuable insights into the mechanisms underlying the anti-inflammatory and anti-tumor effects of PDT, laying a foundation for its clinical application in CRC and IBD treatment and contributing to advancements in intestinal disease therapy.

## Supplementary Material

Supplementary tables.

## Author Contributions

KPW and BYD contributed to conceiving and designing the study. BYD, KPW, LLZ, ZDQ, and WGD performed the article searching. KPW, BYD, LLZ, ZDQ, and WXW extracted the data. BYD and KPW wrote the manuscript. WGD and WXW supervised the manuscript. all authors revised and agree to be accountable for the content of the work.

## Figures and Tables

**Table 1:** The studies related to the effective mitigation of IBD by PDT

Years	Objects	Photosensitizer and concentration	Wavelength (nm)	Light treatment parameters	Main Findings	Refs.
1999	6 UC patient (2 men)	5-ALA 10 or 20 mg/Kg	/	/	Inhibiting inflammatory response	44
2011	T cell-mediated colitis model mice	5-ALA 15mg/Kg	635	5 or 10 J/cm^2^ at 100 mW/cm^2^	Decreasing the proinflammatory cytokines IL-6, IL-17, and INF-γ, and decreasing the number of CD4^+^ T cells	7
2015	AOM/ DSS-induced UC AND CRC model	Foslip 0.1, 0.05 or 0.001 mg/Kg	652	20 J/cm^2^ at 100 mW/cm^2^	Decreasing the expression of a wide range of inflammatory mediators; lowering neutrophil influx; prevents the onset of a dysbiotic microbiota	18
2018	DSS -induced UC model mice	5-ALA	630	10 J/cm^2^ at 300 mW/cm^2^	Regulating Neat1-miRNA204-5p-PI3K-AKT axis	16
2021	TNBS-induced UC model rats	LD4 60,120 240 μg/Kg	650	65 J/cm^2^	Decreasing the expression of IL-6, IL-1, TNFα, MDA, MPO; increasing the expression of GSH and SOD; inhibition of AOC1	19
2022	DSS-induced UC model mice	5-ALA 15mg/Kg	630	10 J/cm^2^ at 300 mW/cm^2^	Regulating miR-301a-3p/ PPARGC1A pathway	38
2023	PBMCs from CD patients	5-ALA 3 mM	630	180 J/cm^2^ at 100 mW/cm^2^	Decreasing survival of CD3-/CD19^+^ B cells; suppressing monocytes; inhibiting subcellular levels of inflammatory cytokines and exosomes	17

AOM: azoxymethane; DSS: dextran sulfate sodium; UC: ulcerative colitis; CD: Crohn's disease; TNBS:2,4,6-trinitrobenzene sulfonic acid; 5-ALA: 5-aminolevulinic acid; MDA: malondialdehyde; MPO: myeloperoxidase; GSH: glutathione; SOD: superoxide oxidase; IL: interleukin; IFN-γ: interferon-gamma; PBMCs: peripheral blood mononuclear cells.

**Table 2:** Summary of representative articles on PDT regulating antitumor effects of CRC

Years	Objects	Photosensitizer and concentration	Wavelength (nm)	Light treatment parameters	Mean Findings	Refs.
PDT inducing apoptosis of CRC						
2007	Human CRC HCT116 cells	PpIX 2mg/mL	632.8	2J/cm^2^	Inducing p53-dependent activation of pro-apoptotic gene expression followed by growth suppression and induction of apoptosis	80
2007	Human CRC HCT116 cells	ATX-S10Na (II) 20 μg/mL	670	2J/cm^2^ at 167 mW/cm^2^	Mediated by p53-Bax network and low levels of Bcl-2 and Bcl-x(L) proteins	79
2010	Human CRC HT29 cells	SiPcGlu 1.5mM	610	48J/cm^2^ at 40 mW/cm^2^	Triggering the apoptotic pathways in both mitochondria and endoplasmic reticulum, but not the lysosome	199
2018	Human CRC LoVo cells	TαPcZn	600-700	53.7J/cm^2^	Direct interaction between p38 MAPK and caspase-9 may regulate mitochondria-mediated apoptosis	50
2018	murine CRC CT26 cells	Pc9-T1107 20 mN	630	2.8J/cm^2^ at 1.17 mW/cm^2^	Lysosomal membrane permeabilization, induction of ER stress, and activation of caspase-dependent apoptotic cell death.	49
PDT regulating antiangiogenic CRC						
2016	Human CRC SW620 and SW480 cells	5-ALA 500, 1000,1500 μM	600-720nm	10, 30, 60 J/cm^2^ at 1.5 mW/cm^2^	5-ALA-PDT markedly reduced VEGF secretion in SW620 cell line, while the ability of SW480 cell line to secrete VEGF remained unchanged	86
2018	Human CRC SW620 and SW480 cells	ALA 10000 μM	600-720nm	10 J/cm^2^ at 1.5 mW/cm^2^	ALA-PDT reduced the release of VEGF in SW620 cell line, and the VEGF secretion levels of SW620 cells were significantly higher than those of SW480 cells	87
PDT activating the immune system of CRC						
2010	CRC BALB/c mouse models expressing b-gal	BPD 1mg/Kg	690	120 J/cm^2^ at 100 mW/cm^2^	The first discovery of the role of antigen expression in PDT immune response.	92
2013	CT26 mouse CRC cells	BPD 1mg/Kg	690	120 J/cm^2^ at 100 mW/cm^2^	Depletion of Treg can enhance the immune response mediated by PDT	93
2016	Human CRC HT29 cells and murine CRC CT26 and MC38 cells	NCP@pyrolipid 2mg/Kg	670	54 J/cm^2^ at 60 mW/cm^2^	PDT combined with anti-PD-L1 inhibited not only the growth of primary CRC but also the distant metastasis of CRC	94
2016	Murine CRC cell CT26 and MC38	NMOFs 1.5mg/Kg	650	90 J/cm^2^ at 100 mW/cm^2^	PDT significantly increases systemic tumor-specific immune response rates to checkpoint blockade cancer immunotherapy	99
2017	mouse CRC CT26 cells	UCNP-Ce6-R837 Nanoparticles	980	500 mW/cm^2^ for 20 mins	The combination of PDT and CTLA-4 checkpoint inhibitors shows a powerful synergistic therapeutic effect, which can not only eliminate primary CRC tumors and inhibit distant metastasis of CRC but also prevent CRC recurrence after treatment	96
2018	mouse CRC CT26 cells	Fe-TBP 0.2 µM	650	100 mW/cm^2^ for 7.5 mins	Combining PDT with anti-PD-L1 treatment can induce the synergistic therapy of CD4^+^ and CD8^+^ cytotoxic T cells for the treatment of CRC	95
2018	mouse CRC CT26 cells	EGFR-CPIG	650	500 mW/cm^2^ for 20 mins	combining PDT with immunotherapy that uses a unique PS targeting EGFR. This approach has precise targeting for CRC and a remarkable inhibitory effect without any recurrence after treatment.	97
2021	mouse CRC CT26 cells	SP_DMC_N 10-40 μg/mL	808	300 mW/cm^2^ for 8 mins	The PS SPDMCN was specifically activated in the tumor microenvironment of CRC, releasing DMC to enhance the effect of PDT	98
2021	HT29 et al.13 human CRC cells and murine CRC MC38 cells	IR700DX-6T 100 or 200 mN	690	18 or 28 J/cm^2^	PDT can inhibit the proliferation of multiple CRC cell lines by inducing ICD	88
PDT activating autophagy of CRC						
2014	PROM1/CD133^+^ CRC cells	PpIX 1 µg/mL	633	1 or 5 J/cm^2^	The elevation of autophagy levels is linked to the enhanced resistance of CSCs to PDT, thus presenting a novel therapeutic strategy for targeting autophagy in the PDT-mediated treatment of CSCs	102
2015	Human CRC SW620 cells	Ce6 2.5mg/ml	650	3 J/cm^2^	Autophagy has potential for cancer treatment, with p38MAPK as a promising therapeutic target for boosting efficacy against CRC	114
2016	Human CRC SW620 cells	5-ALA 3 mM	610-650	4.5 J/cm^2^ at 60 mW/cm^2^	The initial analysis of the impact of PDT on the expression of autophagy-associated proteins Beclin-1, Atg7, and LC3 has improved our understanding of the link between autophagy and PDT	115
2017	Human CRC SW620 and HCT116 cells	PS-II 10 mg/Kg	630	5, 10, 20 J/cm^2^ at 100 mW/cm^2^	combination of PDT with autophagy enhances the inhibitory effect on CRC	112
2017	Human CRC HCT8 and HCT116 cells	Hypericin	630	7.2 J/cm^2^	combination of PDT with autophagy enhances the inhibitory effect on CRC	119
2017	Human CRC CaCo2 and SW480 cells	PpIX	618-652	1, 2, 3, 4, 5 J/cm^2^ at 165 mW/cm^2^	PDT triggers autophagy as a means of survival, and the activation of the novel HIF-1α/VMP1/autophagic pathway may shed light on the resistance of CRC cells to PDT	120
2018	Human CRC HCT116 cells	PpIX 1 mg/ml	630	5 or 10 J/cm^2^	combination of PDT with autophagy enhances the inhibitory effect on CRC	13
2018	Human CRC SW620 cells	Ce6 0.5 μg/ml	650	6 J/cm^2^	combination of PDT with autophagy enhances the inhibitory effect on CRC	116
2020	Human CRC SW480, HCT116, LoVo, and DLD1 cells	Ce6 1.25 mg/Kg	670	4/cm^2^ at 800 mW/cm^2^	overexpressing p62 can instead increase the effectiveness of PDT and CRC cell lines with p62 knocked out were less sensitive to PDT	121
2020	Human CRC SW480 and HCT116	m-THPC and VP (0.375- 12.0 μmol/L)	650	3/cm^2^ at 10 mW/cm^2^	The anti-cancer efficacy of PDT-mediated CRC cells can be significantly reduced by deactivating the ROS/JNK. signaling pathway through the inhibition of autophagy	122
2021	Human CRC SW480	Ce6 0.125 -8 µg/mL	650	6J/cm^2^	combination of PDT with autophagy enhances the inhibitory effect on CRC	117
2022	Human CRC SW620	Ce6 0.5 µg/mL	650	6J/cm^2^	combination of PDT with autophagy enhances the inhibitory effect on CRC	118
PDT regulating miRNAs of PDT						
Years	Objects	PS	miRNAs	Potential Targets	pathways	Refs.
2017	Human CRC HT29, HCT116, and RKO cells	mTHPC	miR-140, miR-30b, miR-3151, miR-506, miR-124, miR-30c, miR-663b	P53	miR124/ iASPP axis	104
2018	Human CRC HT29, HCT116, and LoVo cells	H2TFPC, H2TFPCSGLc	miR-15a, miR-15b, miR-29a, miR-196a, miR-221, miR-25	TNFAIP3	LIFR-AS1/miR-29a/TNFAIP3 axis	136
2020	Human CRC CX-1 cells	DVDMS	miR-7112-3p	PERK	PERK/ATF4/ CHOP/caspase cascade pathway	78
2021	Human CRC SW620, RKO, HCT116, and LoVo cells	5-ALA	miR-124	P53	c-Myc/ Neat1/miR-124/ /iASPP/p53 axis	137

b-gal: b-galactosidase; DMC: immune modulator; Ce6: chlorin e6; m-THPC: Meta-tetra hydroxyphenyl chlorin; VP: verteporfin; DVDMS: Sinoporphyrin sodium; PS- II: Photosan-II;

**Table 3:** Summary of clinical trials and case reports investigating the use of PDT for CRC

Years	Patients	Photosensitizer and concentration	Wavelength (nm)	Light treatment parameters	Research type	Results	Refs.
1991	6 advanced rectal cancer patients, (3 males and 3 females), average 66years old (range 37-91)	Photofrin II, 2 mg/kg body weight	630 ± 3	50-200J/cm^2^	Phase I/II Study	The tumors in two patients exhibited significant destruction, leading to the alleviation of pain and obstruction symptoms in one patient.	146
1994	14CRC patients, (10 men and 7 females),	Hpd, 5 or 3 or111 mg /kg body weight	640	N/A	Phase I/II Study	AIOPDT leads to a reduction in the postoperative recurrence rate among patients with CRC	147
1994	33 patients with Tis or T1 cancers of the gastrointestinal tract	Hpd, 5 mg /kg body weight	632	220 J/cm^2^	Phase II/Ⅲ Study	17 patients were observed to exhibit complete eradication of the localized tumor and negative histopathological findings	148
1995	18 patients with gastrointestinal tumors (12 men and 6 females), average 79years old (range 38-93)	ALA 30-60 mg /kg body weight	628	100J/ cm^2^	a pilot study	ALA-PDT may be a promising technique for the treatment of small gastrointestinal tumors.	149
1995	6 patients with duodenal or rectal tumors	ALA 60 mg /kg body weight or Photofrin 2 mg /kg body weight	630	100J/ cm^2^	a pilot study	PDT is a promising treatment for inoperable polyps in patients with familial adenomatous polyposis	150
1998	22 gastrointestinal tract cancer patients	m-THPc 0.15 mg /kg, Photofrin 2 mg /kg, ALA 60 mg /kg (body weight)	628 for ALA and Photofrin, 650 for mTHPc	50-150 J/cm^2^ for ALA and Photofrin, 10-15 J/cm^2^ for mTHPc	a pilot study	PDT is a promising treatment for small localized tumors in patients unsuitable for surgery	151
2005	24 patients with CRLM	Mthpbc 3mg/Kg or 6mg/Kg (body weight)	740	60 J/cm persist 300 to 600 seconds	Phase Ⅰ Study	PDT can safely and effectively treat unresectable CRLM	152
2019	A 56 years old man with low rectal cancer after ultra-low anterior resection	Porphyrin 2mg/kg body weight	630	100 mw/cm^2^	Case report	PDT successfully treats rectal cancer R1	27

PpIX: Protoporphyrin IX; HPD: hematoporphyrin derivative; AIOPDT: adjuvant intraoperative photodynamic therapy; CRLM: colorectal liver metastasis; m-THPC: Meta-tetra hydroxyphenyl chlorin

## Abbreviations

PDT: Photodynamic therapy
PS: photosensitizer
ROS: reactive oxygen species
HDPDT: High-dose Photodynamic therapy
LDPDT: low-dose Photodynamic therapy
IBD: inflammatory bowel disease
UC: ulcerative colitis
CD: Crohn's disease
CRC: colorectal cancer
CAC: inflammatory bowel disease -related colorectal cancer
TNF-α: tumor necrosis factor-α
CRLM: colorectal liver metastasis
APCs: antigen presenting cells
MHC: major histocompatibility complex
DCs: Dendritic cells
CTLA-4: cytotoxic T-lymphocyte-associated protein 4
COX-2: cyclooxygenase-2
NF-KB: nuclear factor kappa B
F/B: Firmicutes/Bacteroidetes
ICD: immunogenic cell death
ER: endoplasmic reticulum
VEGF: Vascular endothelial growth factor
Treg: T regulatory cells
DMC: immune modulator
CSCs: CRC stem cells
GSH: glutathione
LIP: labile iron pool
TME: tumor microenvironment
Cu-Cy: Copper-cysteamine nanoparticles
LPO: lipid peroxides
MDA: malondialdehyde
AIOPDT: adjuvant intraoperative photodynamic therapy
5-ALA: 5-aminolevulinic acid
BPD: Liposomal benzoporphyrin derivative mono acid ring A
MPO: myeloperoxidase
SOD: superoxide oxidase
IL: interleukin
IFN-γ: interferon-gamma
PBMCs: peripheral blood mononuclear cells
Ce6: chlorin e6
m-THPC: Meta-tetrahydroxyphenylchlorin
VP: verteporfin
DVDMS: Sinoporphyrin sodium
HPD: hematoporphyrin derivative
b-gal: b-galactosidase
AIE: aggregation-induced emission
EVs: extracellular vesicles
