# Supplementary material for: Photodynamic Therapy for Inflammatory and Cancerous Diseases of the Intestines: Molecular Mechanisms and Prospects for Application
Source: Int J Biol Sci. 2023 Sep 4;19(15):4793–810. doi: 10.7150/ijbs.87492 (PMC10539702; doi:10.7150/ijbs.87492)
Supplement: Supplementary file 1 — Supplementary tables. [file ijbsv19p4793s1.pdf]

Table S1 Types of PS and its application on various medical conditions

Type	Characteristic	Examples	Application
First-generation PS	Primarily excited by visible light	hematoporphyrin derivatives and Photofrin	specific cancer treatments
Second-generation PS	Better tumor selectivity, better photodynamic properties, and greater water solubility compared to first-generation PS	ALAand its esters,m-THPC	in the treatment of superficial skin cancers， inflammatory diseases, and pre-cancerous lesions
Third-generation PS	Highly selective, stable, and efficient ROS generation, molecular targeting ability, specific binding to cancer cells	chlorins and phthalocyanines.	promise in targeted cancer therapies and are being extensively studied in preclinical and clinical trials.
Nanoparticle-based PS	Higher solubility, stability, and tumor targeting compared to second-generation PS	Cu-cy, ChitoPEITC,	Cell experiments and animal experiments targeting specific tumors and enhancing PDT
Hybrid PS	Hybrid PS is a combination of different PS or PS with other therapeutic agents, aiming to achieve synergistic effects enhancing PDT efficacy and addressing drug resistance in cancer treatment.	metal-organic frameworks composed of mesoTetra porphine and ferric ion, ICG@MS-rGO-FA	Cell experiments and animal experiments targeting specific cancer recurrence and drug resistance.

ALA: 5-aminolevulinic acid; m-THPC: Meta-tetra hydroxyphenyl chlorin; Cu-Cy: Copper-cysteamine nanoparticles; ChitoPEITC phenethyl-conjugated chitosan oligosaccharide; ICG@MS-rGO-FA: ICG-encapsulated mesoporous silica (MS)-coated rGO nanocomposite.

Table S2 Comparison of the mechanisms of action and therapeutic effects between PDT and the combination of biologic therapy and immunomodulators

Objects	PDT	combination of biologic therapy and immunomodulators
Mechanism of action	Employing PS activated by light to generate ROS, leads to localized tissue damage and cell death. It primarily targets and eliminates abnormal cells, such as inflamed tissues	Biologic therapies focus on specific molecules involved in the inflammatory process, such as TNF-α, interleukins, and integrins. Immunomodulators regulate the immune response to reduce inflammation and control the disease
Cellular effects	Directly damaging cells in the targeted area, inducing apoptosis and cell death. Additionally, it influences the local microenvironment, resulting in reduced inflammation and closure of neovessels	Function by suppressing the overactive immune response responsible for chronic inflammation in IBD and decreasing cytokine production and immune cell activation.

Safety profile	Generally considered safe, with minimal systemic side effects. However, it may cause temporary photosensitivity and localized tissue damage at the treatment site.	This may entail certain risks, including increased susceptibility to infections and potential long-term immunosuppression
Treatment outcome	Promising results in treating IBD. Reducing inflammation, promoting healing, and alleviating symptoms. The existing evidence from clinical research is currently insufficient.	Exhibited efficacy in managing moderate to severe IBD. Inducing remission and sustaining long-term disease control.
Treatment Considerations	Suitable for localized or superficial lesions and may be less effective for extensive or deep-seated inflammation in IBD.	Reserved for patients with moderate to severe IBD who have not responded adequately to conventional treatments
